# AMP-Activated Protein Kinase and Host Defense against Infection

**DOI:** 10.3390/ijms19113495

**Published:** 2018-11-06

**Authors:** Prashanta Silwal, Jin Kyung Kim, Jae-Min Yuk, Eun-Kyeong Jo

**Affiliations:** 1 Department of Microbiology, Chungnam National University School of Medicine, Daejeon 35015, Korea; pst.ktz@gmail.com (P.S.); pcjlovesh6@naver.com (J.K.K.); 2 Infection Control Convergence Research Center, Chungnam National University School of Medicine, Daejeon 35015, Korea; 3 Department of Medical Science, Chungnam National University School of Medicine, Daejeon 35015, Korea; 4 Department of Infection Biology, Chungnam National University School of Medicine, Daejeon 35015, Korea; yjaemin0@cnu.ac.kr

**Keywords:** AMPK, infection, mycobacteria, host defense

## Abstract

5′-AMP-activated protein kinase (AMPK) plays diverse roles in various physiological and pathological conditions. AMPK is involved in energy metabolism, which is perturbed by infectious stimuli. Indeed, various pathogens modulate AMPK activity, which affects host defenses against infection. In some viral infections, including hepatitis B and C viral infections, AMPK activation is beneficial, but in others such as dengue virus, Ebola virus, and human cytomegaloviral infections, AMPK plays a detrimental role. AMPK-targeting agents or small molecules enhance the antiviral response and contribute to the control of microbial and parasitic infections. In addition, this review focuses on the double-edged role of AMPK in innate and adaptive immune responses to infection. Understanding how AMPK regulates host defenses will enable development of more effective host-directed therapeutic strategies against infectious diseases.

## 1. Introduction

5′-AMP-activated protein kinase (AMPK) is an intracellular serine/threonine kinase and a key energy sensor that is activated under conditions of metabolic stress [1,2,3]. It governs a variety of biological processes for the maintenance of energy homeostasis in response to metabolic stresses such as adenosine triphosphate (ATP) depletion [2]. Due to its critical function in metabolic homeostasis, much research has focused on the roles of AMPK in metabolic diseases and cancers [4,5,6]. However, much less is known about the function of AMPK in infection [7]. Due to the energetic demands of infected cells, most infections by intracellular pathogens are associated with activation of host AMPK, presumably to promote microbial proliferation [8]. AMPK functions as a modulator of host defenses against intracellular bacterial, viral, and parasitic infections [9,10,11,12]. Indeed, numerous viruses have the ability to trigger metabolic changes, thereby modulating AMPK activity and substrate selection [13], and AMPK signaling could facilitate or inhibit intracellular viral replication depending on the virus infection [14].

This review focuses on the double-edged role of AMPK in the regulation of host antimicrobial defenses in infections of viruses, bacteria, and parasites. In this review, we describe the existing evidence for the defensive and inhibitory roles of AMPK and the mechanisms underlying its regulation of innate and inflammatory responses. Finally, we describe AMPK-targeting agents that enhance host defenses against infection or control harmful inflammation.

## 2. Overview of AMPK

**Figure 1:**
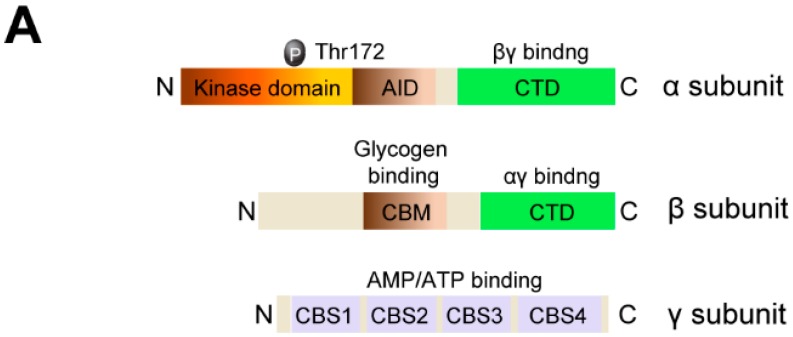

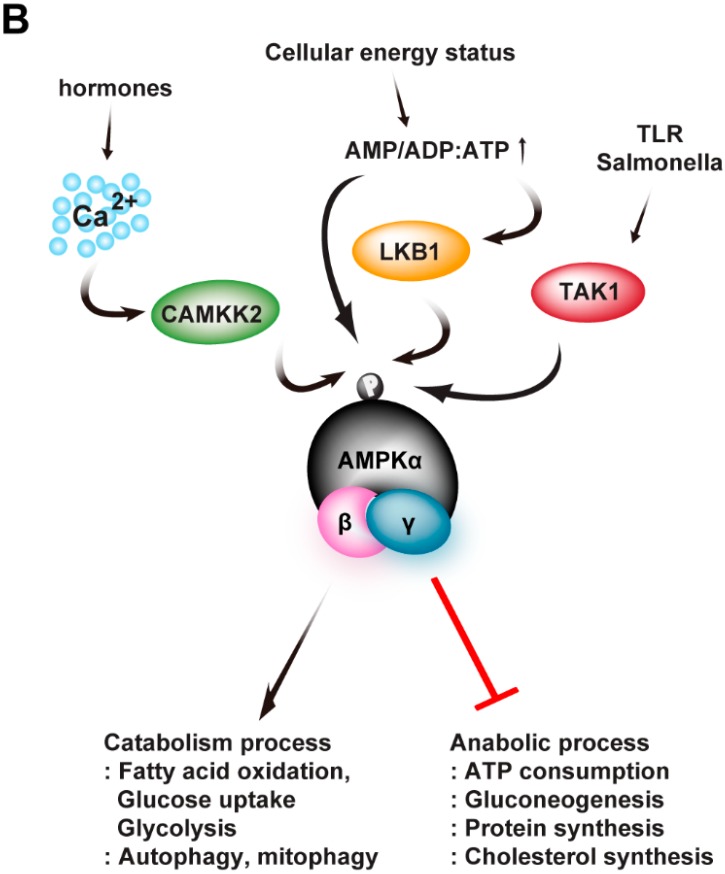
Domain structures of the 5′ AMP-activated protein kinase (AMPK) subunits and the mechanisms that regulate activation of AMPK signaling pathways. (**A**) Conserved domain structure of AMPK subunits consisting of a catalytic α subunit, scaffolding β subunit, and regulatory γ subunit. AID, autoinhibitory domain; CBM, carbohydrate-binding module; CBS, cystathionine-beta-synthase; CTD, C-terminal domain. (**B**) AMPK is activated by the upstream kinases LKB1, CAMKK2 and TAK1 associated with the canonical pathway (triggered by an increased cellular AMP/ATP ratio) or the non-canonical pathway (triggered by an increased intracellular Ca^2+^ concentration or infection/TLR activation). Activated AMPK modulates cellular homeostasis, such as energy metabolism and autophagy, and mitochondrial homeostasis. (black arrow indicate activation/increase; bar-headed red arrow indicates inhibition/decrease). CAMKK2, calcium/calmodulin-dependent kinase kinase 2; LKB1, liver kinase B1; TAK1, Transforming growth factor-β-activated kinase 1; TLR, Toll-like receptor.

5′-AMP-activated protein kinase (AMPK), a serine/threonine kinase, is a key player in bioenergetic homeostasis to preserve cellular ATP [1]. AMPK is activated in response to an increased cellular adenosine monophosphate (AMP)/ATP or adenosine diphosphate (ADP)/ATP ratio, thus promoting catabolic pathways and suppressing biosynthetic pathways [1,2,3]. Mammalian AMPK exists as a heterotrimeric complex comprising a catalytic subunit α (α1 and α2), a scaffolding β subunit (β1 and β2), and a regulatory γ subunit (γ1, γ2, and γ3) (Figure 1A) [15]. Multiple isoforms of AMPK are encoded by distinct genes of the subunit isotypes, depending on the cell/tissue or species [16]. The AMPK subunit composition and ligand-induced activities of each AMPK isoform complex can differ among cell types, although the α1, β1, and γ1 isoforms are ubiquitously expressed [16,17].

The AMPK α subunit contains a kinase domain at the N terminus, which is activated by phosphorylation of Thr-172 by the major upstream liver kinase B1 (LKB1) [18,19]. In contrast to LKB1, the upstream Ca^2+^-calmodulin-dependent kinase kinase (CaMKK) activates AMPK in response to an increased intracellular Ca^2+^ concentration in the absence of significant changes in ATP/ADP/AMP levels [20]. The regulatory β subunit of AMPK contains a glycogen-binding domain that can sense the structural state of glycogen [21]. Four consecutive cystathionine-β-synthase domains in the regulatory γ subunit are essential for binding to adenosine nucleotides to form an active αβγ complex (Figure 1B) [22,23].

Different AMPK isoforms may have distinct biological functions in different physiological and pathological systems. AMPK governs the cellular energy status by acting as a crucial regulator of energy homeostasis in response to various metabolic stresses, including starvation, hypoxia, and muscle contraction. AMPK activity can be altered by numerous factors, including hormones, cytokines, and nutrients, as well as diverse pathological changes such as metabolic disturbances [24,25]. Because AMPK is important in the adaptation to energy stress, dysregulation of or decreased AMPK activation is implicated in the development of metabolic disorders associated with insulin resistance [6]. In addition to its primary role in the regulation of energy metabolism, AMPK signaling plays a critical role in host–microbial interactions [7]. Furthermore, infections by several viruses result in dysregulation or stimulation of AMPK activity [13]. In mycobacterial infections, AMPK activation promotes activation of host defenses in macrophages and in vivo [12,26]. However, much less is known about the function of AMPK in innate host defenses compared with that in the regulation of metabolism and its mitochondrial function.

## 3. Multifaceted Role of AMPK in Antimicrobial Responses

**Figure 2:**
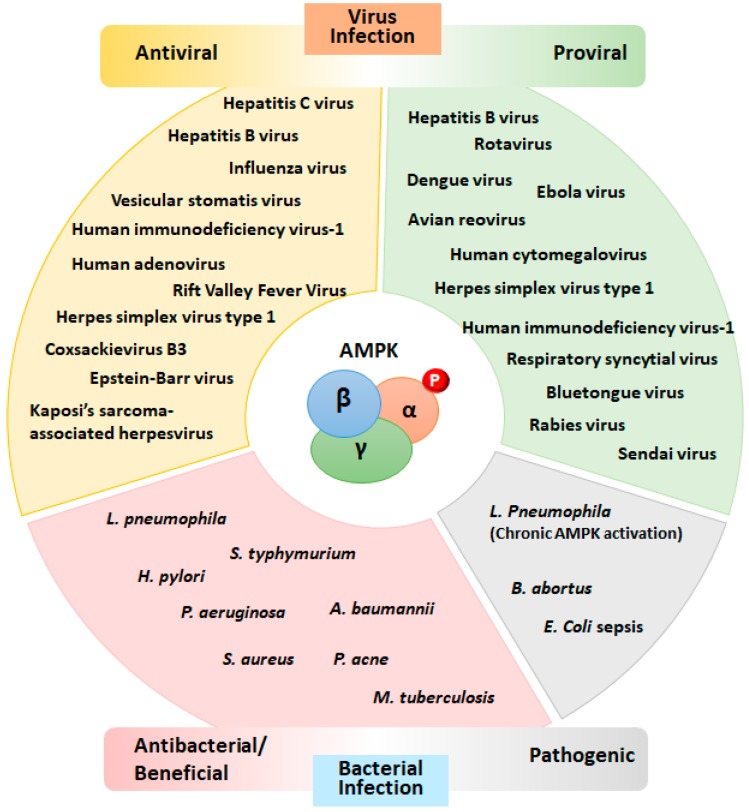
Multifaceted roles of AMPK in viral and bacterial infections. A variety of viruses and bacteria modulate host AMPK activity to promote their growth in host cells. Activation of the AMPK signaling pathway has been implicated in both beneficial antiviral (**left upper**) and detrimental proviral (**right upper**) responses. In addition, AMPK activation promotes the host response to infections by various bacteria (**left lower**) but, in some cases, promotes a detrimental response (**right lower**). The detailed mechanisms by which AMPK activation/inhibition affects infection outcomes are listed in Table 1, Table 2, Table 3 and Table 4.

Viruses have evolved strategies to manipulate the AMPK signaling pathway to escape host defenses. Indeed, several pathogens can modulate the activity of AMPK/mTOR to obtain sufficient energy for their growth and proliferation [8]. In this review, we discuss microbial manipulation of AMPK activity to affect host defenses against infections. Figure 2 summarizes the multiple roles of AMPK in the viral and bacterial infections addressed in this review. The detailed mechanisms and outcomes of host–pathogen interactions in terms of AMPK modulation are described in Table 1, Table 2, Table 3 and Table 4.

### 3.1. Roles of AMPK in Viral Infections

#### 3.1.1. Beneficial Effects of AMPK on Virus Infections

Hepatitis C virus (HCV) is a major etiologic agent of chronic liver disease worldwide. HCV infection inhibits AMPKα phosphorylation and signaling [27], and the AMPK agonist metformin suppresses HCV replication in an autophagy-independent manner [28]. Moreover, HCV core protein increases the levels of reactive oxygen species (ROS) and alters the NAD/NADH ratio to decrease the activity and expression of sirtuin 1 (SIRT1) and AMPK, thereby altering the metabolic profile of hepatocytes. This mechanism is implicated in the pathogenesis of hepatic metabolic diseases [29]. As AMPK is a crucial regulator of lipid and glucose metabolism, pharmacological restoration of AMPK activity inhibits lipid accumulation and viral replication in HCV-infected cells [27]. In addition, metformin enhances type I interferon (IFN) signaling by activating AMPK, resulting in inhibition of HCV replication [30]. AMPK inhibition resulted in the downregulation of type I IFN signaling and rescue of the metformin-mediated decrease in the HCV core protein level [30]. Moreover, the AMPK activator 5-aminoimidazole-4-carboxamide 1-β-d-ribofuranoside (AICAR) inhibits HCV replication by activating AMPK signaling, although the anti-HCV effect of metformin is independent of AMPK activation [31]. In chronic HCV infection, the expression of Sucrose-non-fermenting protein kinase 1/AMP-activated protein kinase-related protein kinase (SNARK), an AMPK-related kinase, is increased to promote transforming growth factor β signaling, which is critical for hepatic fibrogenesis [32]. A more recent study showed that HCV-mediated ROS production triggers AMPK activation to attenuate lipid synthesis and promote fatty acid β-oxidation in HCV-infected cells [33]. These data suggest that HCV inhibits AMPK activation to promote its replication, and that the restoration of AMPK activity may be an effective therapeutic modality for HCV infection that acts by metabolic reprogramming or modulation of type I IFN production in host cells [27,28,30,33].

In hepatitis B viral (HBV) infection, AMPK can promote or inhibit viral replication. The detrimental effects of AMPK are described in the following section. Xie et al. reported that AMPK, which is activated by HBV-induced ROS accumulation, suppresses HBV replication [9]. Mechanistically, AMPK activation leads to HBV-mediated autophagic activation, which enhances autolysosome-dependent degradation to restrict viral proliferation [9]. AMPK activity is also involved in the defense against vesicular stomatitis virus, the causal agent of an influenza-like illness, by activating stimulator of IFN genes (STING) [10]. Treatment of mouse macrophages or fibroblasts with an AMPK inhibitor suppressed the production of type I IFN and TNF-α in response to a STING-dependent ligand or agonist, suggesting a role for AMPK in STING signaling [10]. AMPK plays a role in the excessive inflammatory cytokine/chemokine levels in Mint3/Apba3 depletion models of severe pneumonia due to influenza virus [34]. Indeed, food-derived polyphenols, such as epigallocatechin gallate and curcumin, are useful for controlling viral and bacterial infections [35]. Although a review of AMPK-modulating polyphenols is beyond our scope, we highlight the therapeutic promise of polyphenols against infection. For example, curcumin from *Curcuma longa* inhibits influenza A viral infection in vitro and in vivo, at least in part by activating AMPK [36]. The polyphenol epigallocatechin gallate attenuates Tat-induced human immunodeficiency virus (HIV)-1 transactivation by activating AMPK [37]. Further studies should examine the ability of food-derived polyphenols to activate AMPK signaling to control viral replication in host cells. 

Human adenovirus type 36, which is associated with obesity, inhibits fatty acid oxidation and AMPK activity and increases accumulation of lipid droplets in infected cells [38]. The AMPK signaling pathway and its upstream regulator LKB1 repress replication of the bunyavirus Rift Valley Fever virus (RVFV), a re-emerging human pathogen [39]. The mechanisms of the antiviral effects of AMPK on RVFV and other viruses are mediated by AMPK inhibition of fatty acid synthesis [39]. Pharmacologic activation of AMPK suppresses RVFV infection and reduces lipid levels by inhibiting fatty acid biosynthesis [39]. In addition, the AMPK/Sirt1 activators resveratrol and quercetin significantly reduce the viral titer and gene expression, as well as increase the viability of infected neurons, in herpes simplex virus type 1 (HSV-1) infection [40]. Moreover, coxsackievirus B3 (CVB3) infection triggers AMPK activation, which suppresses viral replication in HeLa and primary myocardial cells [41]. The AMPK agonists AICAR and metformin suppress CVB3 replication and attenuate lipid accumulation by inhibiting lipid biosynthesis [41]. Thus, regulation of fatty acid metabolism by AMPK signaling is an essential component of cell autonomous immune responses [39]. 

Latent membrane protein 1 (LMP1) of Epstein-Barr virus (EBV) inactivates LKB1/AMPK, whereas AMPK activation by AICAR abrogated LMP1-mediated proliferation and transformation of nasopharyngeal epithelial cells, suggesting therapeutic potential for EBV-associated nasopharyngeal carcinoma [42]. Moreover, constitutive activation of AMPK inhibited lytic replication of Kaposi’s sarcoma-associated herpesvirus in primary human umbilical vein endothelial cells [43]. These data suggest that AMPK suppresses cell transformation and infection-related tumorigenesis in a context-dependent manner. The roles of AMPK in viral infection are listed in Table 1.

#### 3.1.2. Detrimental Effects of AMPK on Virus Infections

Several viruses manipulate AMPK signaling to promote their replication. Genome-scale RNA interference screening of host factors in rotaviral infection identified AMPK as a critical factor in the initiation of a rotavirus-favorable environment [44]. In dengue viral infections, the 3-hydroxy-3-methylglutaryl-CoA reductase (HMGGR) activity elevated by AMPK inactivation resulted in generation of a cholesterol-rich environment in the endoplasmic reticulum, which promoted formation of viral replication complexes [45]. Also, dengue viral infection stimulates AMPK activation to induce proviral lipophagy, thereby enhancing fatty acid β-oxidation and viral replication [46].

In HBV infection, the HBV X protein activates AMPK, and inhibition of AMPK reduces HBV replication in rat primary hepatocytes [47]. Inhibition of AMPK led to activation of mTORC1, which is required for inhibition of HBV replication in the presence of low AMPK activity [47]. The crosstalk between AMPK and mTORC1 may enable development of therapeutics that suppress HBV replication and thus also hepatocellular carcinoma (HCC) development [47]. However, as described in Section 3.1.1, AMPK activation by AICAR inhibits extracellular HBV production in HepG2 cells [9]. The discrepancy may be attributed to the use of different cell lines in the two studies [9,47]. Further work should address the role of AMPK in HBV infection in vitro and in vivo. In infection by Zaire Ebolavirus (EBOV), the expression levels of the γ2 subunit of AMPK are correlated with EBOV transduction in host cells. In mouse embryonic fibroblasts treated with a small-molecule inhibitor of AMPK (compound C), it was shown that AMPK activity is required for EBOV replication in host cells and EBOV glycoprotein-mediated entry/uptake [48]. In addition, Avian reoviral infection upregulates AMPK phosphorylation, which leads to activation of mitogen-activated protein kinase (MAPK) p38 in Vero cells, which enhances viral replication [49]. The nonstructural protein p17 of avian reovirus positively regulates AMPK activity, inducing autophagy and increasing viral replication [50].

In HSV-1 infection, the activated AMPK/Sirt1 axis inhibits host-cell apoptosis during early-stage infection, which promotes viral latency and protects neurons [51]. However, during the later stages of infection, HSV-1 induces apoptosis of host cells concomitantly with Sirt1 activation [51]. In HIV-infected cocaine abusers, AMPK signaling plays a role in energy deficit and neuronal dysfunction, which are associated with the development of neuroAIDS [52]. These data suggest that differential regulation of AMPK signaling is a determinant of the viral infection course.

Using a kinome-profiling approach, AMPK and related kinases were found to be effectors of human cytomegalovirus (HCMV) replication [53,54]. HCMV infection induces AMPK and CaMKK2 (upstream activator of AMPK)-dependent remodeling of core metabolism, both of which are required for optimal yield and replication of HCMV [53,54]. Notably, inhibition of AMPK activity by short-interfering RNA-mediated AMPK knockdown or an AMPK antagonist (compound C) prevents viral gene expression, providing valuable insight into the mechanisms of HCMV infection [53]. In addition, the AMPK activation-dependent modulation by HCMV of host-cell metabolism is associated with HCMV replication [55]. HCMV-mediated AMPK activation is dependent on CaMKK, and inhibition of AMPK activity abrogated HCMV replication and DNA synthesis [55]. Furthermore, the cardiac glycoside digitoxin induces phosphorylation of AMPK/ULK1, whereas it suppresses mTOR activity to increase autophagic flux and inhibit HCMV replication [56]. Moreover, HCMV induces production of the host protein viperin [57], which is required for AMPK activation, transcriptional activation of GLUT4 and lipogenic enzymes, and lipid synthesis [58]. The enhanced lipid synthesis promotes formation of the viral envelope and production of HCMV virions [58].

In infection, host-cell autophagy plays an important role in host defense and virus survival. Several viruses can manipulate or subvert the autophagic machinery to favor viral replication. For example, respiratory syncytial virus activates autophagy via the AMPK/mTOR signaling pathway to enhance its replication by inhibiting host-cell apoptosis [59]. Bluetongue virus, a double-stranded segmented RNA virus, also induces host-cell autophagy by activating AMPK [60]. Moreover, AMPK is an upstream regulator of rabies virus-induced incomplete autophagy to provide the scaffolds for viral replication [61]. In Sendai viral infection, AMPK activity is required for autophagic initiation to promote viral replication [62]. In oncogenic EBV infection, the increased cell survival caused by AMPK-mediated autophagic activation maintains early hyperproliferation of infected cells [63]. These data suggest AMPK activity to be a therapeutic target for the development of novel antiviral agents.

Importantly, type I IFN, a critical effector in the antiviral response, attenuates AMPK phosphorylation and increases the intracellular ATP level [64]. In addition, IFN-β-mediated glycolytic metabolism is important for the acute phase of the antiviral response to CVB3 [64]. The antiviral cytokine IFN-β regulates host-cell metabolism to enhance glucose uptake and ATP generation, which promote the antiviral response [64]. In infection by snakehead vesiculovirus, miR-214 targeting AMPK suppressed viral replication and upregulated IFN-α expression [65]. Thus, regulation of AMPK activity by the host–pathogen interaction mediates diverse metabolic effects, which modulate viral replication and the host defense response. The beneficial and detrimental effects of AMPK on viral infections are summarized in Table 1 and Table 2, respectively.

### 3.2. Bacterial Infections and AMPK Activation

Intracellular pathogens manipulate the AMPK signaling pathway to alter their metabolic environment to favor bacterial survival or pathogenesis. Mitochondrial dysfunction triggers AMPK signaling, thus enhancing the proliferation of *Legionella pneumophila*, a respiratory pathogen, in *Dictyostelium* cells [66]. Inhibition of AMPK activation reversed the increased *Legionella* proliferation in host cells with mitochondrial disease [66]. However, the AMPK activator metformin triggers mitochondrial ROS generation and activates the AMPK signaling pathway to enhance the host response to *L. pneumophila* in macrophages and promote survival in a murine model of *L. pneumophila* pneumonia [67]. Thus, the role of AMPK activation in bacterial infections differs depending on the host species.

*Salmonella typhimurium* degrades SIRT1/AMPK to evade host xenophagy [68]. In addition, cytosolic *Salmonella* is ubiquitinated and targeted for xenophagy by AMPK activation [69,70]. AMPK activation by AICAR induces autophagy and colocalization of *Salmonella*-containing vacuoles with LC3 autophagosomes [68], whereas inhibition of AMPK by compound C increases bacterial replication by suppressing autophagy [69]. In *Salmonella*-infected cells, AMPK activation is mediated through toll-like receptor-activated TGF-β-activated kinase 1 (TAK1) [69] which is a direct upstream kinase of AMPK in addition to LKB1 and CaMKK2 [71]. In *Brucella abortus* infection, AMPK activation enhances intracellular growth of *B. abortus* by inhibiting nicotinamide adenine dinucleotide phosphate (NADPH) oxidase-mediated ROS generation [72]. In models of *Escherichia coli* sepsis, ATP-induced pyroptosis is blocked by piperine, a phytochemical present in black pepper (*Piper nigrum* Linn) [73]. The inhibitory effects of piperine on pyroptosis and systemic inflammation are mediated by regulation of the AMPK signaling pathway, as shown by suppression of ATP-mediated AMPK activation by piperine treatment in vitro and in vivo [73]. These data suggest that AMPK plays multiple roles in bacterial infections.

AMPK activation promotes host defenses against infections by several microbes. Transcriptomic and proteomic analyses of a *Caenorhabditis elegans* model indicated that AMPKs function as regulators and mediators of the immune response to infection by, for example, *Bacillus thuringiensis* [74]. Several small-molecule AMPK activators exert protective effects against *Helicobacter pylori*-induced apoptosis of gastric epithelial cells [70,75]. The AMPK agonists A-769662 and resveratrol, as well as AMPKα overexpression, inhibit apoptosis in *H. pylori*-infected gastric epithelial cells [76]. The AMPK activator compound 13 ameliorates *H. pylori*-induced apoptosis of gastric epithelial cells by modulating ROS levels via the AMPK-heme oxygenase-1 axis [76]. Blockade of AMPK signaling significantly abrogates the protective effect of compound 13 against *H. pylori* within gastric epithelial cells [76]. 

Metformin and AICAR repress infection by *Pseudomonas aeruginosa*, an important opportunistic pathogen, of airway epithelial cells by inhibiting bacterial growth and increasing transepithelial electrical resistance [77]. AMPKα1 depletion increased the susceptibility to *Staphylococcus aureus* endophthalmitis in mice [78], suggesting a protective role for AMPK in bacterial retinal inflammation. Moreover, AMPK activation by AICAR enhances its anti-inflammatory effects, phagocytosis, and bactericidal activity against *S. aureus* infection of various phagocytic cells including microglia, macrophages, and neutrophils [78]. Epigallocatechin gallate, a polyphenol in green tea, inhibits the viability of *Propionibacterium acnes*, a pathogen associated with acne, and exerted an antilipogenic effect in SEB-1 sebocytes by activating the AMPK/sterol regulatory element-binding protein pathway [35]. Moreover, *Acinetobacter baumannii*, an emerging opportunistic pathogen, activates autophagy via the AMPK/ERK/mTOR pathway to promote an antimicrobial response to intracellular *A. baumannii* [79]. The roles of AMPK in bacterial infection are summarized in Table 3.

### 3.3. Roles of AMPK in Mycobacterial Infection

The seminal study by Singhal et al. addressed the effect of metformin as an adjunctive therapy for tuberculosis. Importantly, metformin suppressed the intracellular growth of *Mycobacterium tuberculosis* (Mtb) in vitro, including drug-resistant strains, by activating the AMPK signaling pathway [80]. In vivo, metformin attenuated the immunopathology and enhanced the immune response and showed a synergistic effect with conventional anti-TB drugs in Mtb-infected mice [80]. The microbicidal effect of metformin in macrophages is due, at least in part, to mitochondrial ROS generation, which is associated with AMPK signaling [80]. Type 2 diabetes mellitus (DM) is re-emerging as a risk factor for human tuberculosis, thus candidate host directed therapeutic targets for tuberculosis combined with DM should be identified [85]. Human cohort studies showed that metformin treatment for DM is associated with a decreased prevalence of latent tuberculosis compared with alternative DM treatments, suggesting metformin to be a candidate HDT for tuberculosis patients with type 2 DM [80,85]. Indeed, Mtb infection inhibits AMPK phosphorylation but increases mTOR kinase activation in macrophages [12]. The AMPK activator AICAR via autophagic activation enhances phagosomal maturation and antimicrobial responses in macrophages in Mtb infection [12]. In human monocytes/macrophages, vitamin D-mediated antimicrobial responses are mediated by the antimicrobial peptide LL-37 via AMPK activation [81]. In addition, LL-37-induced autophagy by phenylbutyrate, alone or in combination with vitamin D, promotes intracellular killing of Mtb in human macrophages via AMPK- and PtdIns3K-dependent pathways [82]. Recent findings revealed the role of gamma-aminobutyric acid (GABA) in AMPK activation to enhance the autophagy and the antimicrobial responses [83]. Silencing of AMPK by a lentiviral short hairpin RNA (shRNA) specific to AMPK reduces GABA-induced autophagic activation as well as phagosomal maturation during Mtb infection [83].

MicroRNAs are small non-coding RNAs involved in the regulation of diverse physiological and pathological processes, including Mtb infection. Mycobacterial infection of macrophages upregulates miR-33 and miR-33*, which target and suppress AMPKα [86]. Interestingly, miR-33/miR-33* regulates autophagy by suppressing AMPK-dependent activation of the transcription of autophagy- and lysosome-related genes and promoting accumulation of lipid bodies in Mtb infection [86]. Mtb infection increases the expression of MIR144*/has-miR-144-5p, which targets DNA damage regulated autophagy modulator 2 (DRAM2), to inhibit the antimicrobial responses to Mtb infection in human monocytes/macrophages. In contrast, autophagic activators enhance production of the autophagy-related protein DRAM2 by activating the AMPK signaling pathway; this contributes to host defenses against Mtb in human macrophages [87].

Although AMPK may play a protective role in tuberculosis, it has also been reported to exert an immunopathological effect by driving the secretion of neutrophil matrix metalloproteinase-8 (MMP-8), resulting in matrix destruction and cavitation, which enhance the spread of Mtb [84]. Neutrophil-derived MMP-8 secretion is upregulated in Mtb infection and neutrophils from AMPK-deficient patients express lower levels of MMP-8, suggesting a key role for MMP-8 in tuberculosis immunopathology [84]. Because the pathogenesis of tuberculosis is complex, further information on the function of AMPK in the immune response to Mtb infection is needed for development of improved therapeutic strategies [88]. The roles of AMPK in mycobacterial infection are listed in Table 3.

### 3.4. Roles of AMPK in Parasite Infections

The immune response to parasitic helminths involves M2-type cells, CD4(+) Th2 cells, and group 2 innate lymphoid cells. AMPK activation regulates type 2 immune responses and ameliorates lung injury in response to hookworm infections [89]. Mice deficient in AMPK α1 subunit exhibited impaired type 2 responses, an increased intestinal worm burden, and exacerbated lung injury [89]. In *Leishmania*-infected macrophages, *Leishmania infantum* causes a metabolic switch to enhance oxidative phosphorylation by activating LKB1/AMPK and SIRT1 [90]. Impairment of metabolic reprogramming by SIRT1 or AMPK suppresses intracellular growth of the parasite, suggesting a role for AMPK/SIRT1 in intracellular proliferation of *L. infantum* [90]. In *Schistosoma japonicum* egg antigen (SEA)-mediated autophagy, which is modulated by IL-7 and the AMPK signaling pathway, ameliorate liver pathology, suggesting AMPK to be a therapeutic target factor for schistosomiasis [91].

Notably, host AMPK activity is decreased by hepatic *Plasmodium* infection. Activation of the AMPK signaling pathway by AMPK agonists, including salicylate, suppresses the intracellular replication of malaria parasites, including that of the human pathogen *Plasmodium falciparum* [92]. These data suggest that host AMPK signaling is a therapeutic target for hepatic *Plasmodium* infection [92]. In addition, resveratrol protects cardiac function and reduces lipid peroxidation and trypanosomal burden in the heart by activating AMPK, suggesting a role for AMPK in Chagas heart disease [93]. The roles of AMPK in parasitic infections are listed in Table 4.

### 3.5. AMPK in Fungal Infection

A recent phosphoproteomic analysis of *Cryptococcus neoformans* (Cn) infection showed that AMPK activation is triggered by fungal phagocytosis and is required for autophagic induction. Interestingly, AMPK depletion in monocytes promoted host resistance to fungal infection in mouse models, suggesting that AMPK represses the immune response to *Cryptococcus* infection [94].

## 4. Roles of AMPK in Innate and Adaptive Immune Responses

**Figure 3:**
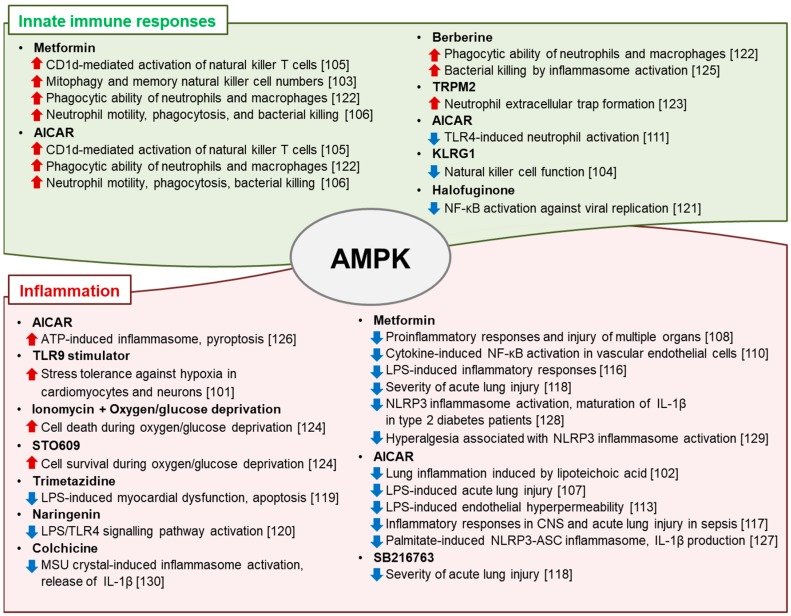
Regulatory effects and underlying mechanisms of small-molecule AMPK activators on the innate immune and inflammatory responses. (red upward arrows indicate activation/increase and blue downward arrows indicate inhibition/decrease)

The roles of AMPK in modulation of the mitochondrial network and energy metabolism, which are associated with the immune response, have been investigated [95]. Here, we briefly review recent data on AMPK regulation of innate and adaptive immune responses in infection and inflammation. Figure 3 summarizes the regulatory roles and mechanisms of a variety of small-molecule AMPK activators in terms of innate immune and inflammatory responses.

The data must be interpreted cautiously, as the small-molecule activators (e.g., AICAR, metformin, and compound C) function via off-target mechanisms, such as AMPK-independent pathways or inhibition of protein kinases other than AMPK [31,96,97]. The beneficial effects of these compounds remain to be fully determined. Thus, selective compounds such as MK-8722 [98] and SC4 [99] and the selective inhibitor SBI-0206965 [100] should be considered for future AMPK-targeted treatment strategies.

### 4.1. Role of AMPK in Regulation of the Innate Immune Response

AMPK is involved in regulation of the innate immune response. For example, the innate-immune stimulator toll-like receptor (TLR) 9 inhibits energy substrates (intracellular ATP levels) and activates AMPK, which enhances stress tolerance in cardiomyocytes and neurons, while stimulation by the TLR9 ligand induces inflammation [101]. The AMPK activator AICAR suppresses the lung inflammation induced by lipoteichoic acid, a major component of the cell wall of Gram-positive bacteria [102]. Natural killer (NK) cells are crucial in the innate immune response to viral infections and transformed cells. Activation of the AMPK signaling pathway or inhibition of mTOR is associated with enhanced mitophagy and an increased number of memory NK cells in antiviral responses [103]. In contrast, increased expression of the inhibitory killer cell lectin-like receptor G1 in aged humans is related to AMPK activation, which has been implicated in disruption of NK cell function [104]. In addition, AMPK activation contributes to CD1d-mediated activation of NK T cells, an important cell type in the innate immune response [105]. The findings above suggest that AMPK plays a pleiotropic role in the regulation of the innate immune response depending on the stimulus and cell type in question.

### 4.2. AMPK Regulation of Local and Systemic Inflammation

AMPK activators enhance neutrophil chemotaxis, phagocytosis, and bacterial killing to protect against peritonitis-induced sepsis [106]. Indeed, AMPK activators including metformin inhibit injurious inflammatory responses, including neutrophil proinflammatory responses and injury to multiple organs such as the lung, liver, and kidney [107,108,109]. Pharmacologic activation of AMPK by metformin, berberine, or AICAR dampens excessive TLR4/NF-κB signaling, M2-type macrophage polarization, and the production of proinflammatory mediators in vitro and in models of sepsis [110,111,112,113,114,115]. The anti-inflammatory effect of metformin in mice with lipopolysachharide (LPS)-induced septic shock and in ob/ob mice is mediated at least in part by AMPK activation [116]. In septic mice, AMPK activation by AICAR or metformin reduces the severity of sepsis-induced lung injury, enhances AMPK phosphorylation in the brain, and attenuates the inflammatory response [117,118].

Treatment with trimetazidine protects against LPS-induced myocardial dysfunction, exerts an anti-apoptotic effect, and attenuates the inflammatory response due to its effect on the SIRT1/AMPK pathway [119]. Moreover, the flavonoid naringenin dampens inflammation in vitro and protects against murine endotoxemia in vivo; these effects are mediated by AMPK/ATF3-dependent inhibition of the TLR4 signaling pathway [120]. In severe acute HBV infection, halofuginone, a plant alkaloid, inhibits viral replication by activating AMPK-mediated anti-inflammatory responses [121]. AMPK activation also enhances the phagocytic capacities of neutrophils and macrophages [122]. Transient receptor potential melastatin 2, an oxidant sensor cation channel, promotes extracellular trap formation by neutrophils via the AMPK/p38 MAPK pathway, enhancing their antimicrobial activity [123]. AMPK activation not only modulates the acute inflammatory response but also promotes neutrophil-dependent bacterial uptake and killing [106].

In perinatal hypoxic–ischemic encephalopathy, prolonged activation of AMPK signaling suppresses the response to oxygen/glucose deprivation and promotes neonatal hypoxic–ischemic injury [124]. Although AMPK inhibition increases neuronal survival, blockade of AMPK prior to oxygen/glucose deprivation increases cell damage and death [124]. Therefore, the clinical implications of AMPK activation are complex, and further preclinical and clinical data are needed to enable therapeutic use of AMPK activators in patients with acute or chronic inflammation.

### 4.3. Role of AMPK in Inflammasome Activation

AMPK is implicated in modulation of NLRP3 inflammasome activation. The bactericidal activity of the isoquinoline alkaloid berberine exerts a bactericidal effect by augmenting inflammasome activation via AMPK signaling [125]. However, metformin increases mortality of mice with bacteremia, likely via an AMPK-mediated increase in ATP-induced inflammasome activation and pyroptosis [126].

AMPK is implicated in the inhibition of palmitate-induced inflammasome activation [127]. The AMPK activator AICAR inhibits palmitate-induced activation of the NLRP3 inflammasome and IL-1β secretion by suppressing ROS generation [127]. In addition, NLRP3 inflammasome activation and production of IL-1β are upregulated in the peripheral mononuclear cells of drug-naïve type-2 diabetic patients, suggesting a role of the inflammasome in the pathogenesis of type-2 diabetes [128]. Interestingly, AMPK activation is responsible for the significantly reduced mature IL-1β level in peripheral myeloid cells from type-2 diabetic patients after two months of metformin therapy [128]. In a model of hyperalgesia, which is associated with NLRP3 inflammasome activation, metformin attenuated the clinical symptoms and improved the biochemical parameters, whereas blockade of AMPK activation by compound C provoked hyperalgesia and increased the levels of IL-1β and IL-18 [129]. Furthermore, pharmacological activation of AMPK inhibits the monosodium urate (MSU) crystal-induced inflammatory response, suggesting a role for AMPK in gouty inflammation. Moreover, colchicine, an inhibitor of microtubule assembly used to treat gouty arthritis, enhances AMPKα-mediated phosphorylation, thereby inhibiting inflammasome activation and IL-1β release [130]. Further studies on the efficacy of AMPK activators against inflammasome-associated diseases are thus warranted.

### 4.4. Role of AMPK in the Regulation of the Adaptive Immune Responses

AMPKα1 is a key regulator of the adaptive immune response, particularly T helper (Th1) 1 and Th17 cell differentiation and the T-cell responses to viral and bacterial infections [131]. In addition, in models of simian immunodeficiency viral infection, AMPK activation is associated with the virus-specific CD8(+) cytotoxic T-lymphocyte population and control of Simian Immunodeficiency Virus (SIV) [132]. The mechanism(s) by which AMPK signaling activates innate and adaptive immune responses and controls excessive inflammation must be determined if the potential of AMPK-targeted therapy is to be realized.

## 5. Conclusions

Although much research has focused on the role of AMPK in the regulation of mitochondrial and metabolic homeostasis, several issues remain to be addressed. Further work should focus on the mechanism(s) by which AMPK modulates host defenses against infections in vivo. Several pathogens modulate the host metabolic environment to promote their survival and replication. Because of its role in regulating mitochondrial metabolism, dynamics, and biogenesis, AMPK signaling can provide energy to the pathogen and/or host, benefitting either. Stimulation of AMPK activity enhances host defenses against diverse viruses, bacteria, and parasites, notably Mtb. Moreover, AMPK links the innate and adaptive immune responses to infection. However, the molecular mechanisms underlying AMPK regulation of innate and adaptive immunity are unclear. AMPK-targeted small molecules have potential as antimicrobial agents as well as metabolic drugs. Further work is needed to enable development of therapeutics that target AMPK to control inflammation and promote host defenses against infection. This work should focus on elucidating the mechanisms by which AMPK and/or AMPK-targeting compounds modulate host defenses against infection.

## Figures and Tables

**Table 1:** Beneficial Effects of AMPK in viral infection.

Pathogen	Small Molecules/Chemicals	Agonist/Antagonist	Involvement of AMPK	Outcome (In Vitro/In Vivo)	Ref.
Hepatitis C virus (HCV)	HCV	-	HCV infection inhibit AMPKα phosphorylation and Akt-TSC-mTORC1 pathway	AMPK inhibition is required for HCV replication (in vitro)	[27,28]
	AICAR, Metformin, A769662	Agonist	Restoration of AMPKα activity	Antiviral effects (in vitro)	[27,28]
	Metformin	Agonist	Type I interferon signaling through AMPK pathway activation	Inhibits HCV replication (in vitro)	[30]
	AICAR	Agonist	AMPK activation (Indirect effects counteracted by compound C)	Suppression of HCV replication (in vitro)	[31]
Hepatitis B virus (HBV)	HBV	-	ROS-dependent AMPK activation in HBV-producing cells	Negatively regulates HBV production	[9]
	AICAR constitutive active AMPKα	Agonist	AMPK activation, autophagic flux activation	Inhibits HBV production (in vitro)	[9]
	Compound C dominant-negative AMPKα	Antagonist	AMPK inhibition	Enhances HBV production (in vitro and in vivo)	[9]
Vesicular stomatitis virus (VSV)	AICAR	Agonist	STING-dependent signaling activation	Type I IFN production and antiviral responses (in vitro)	[10]
	Compound C	Antagonist	Inhibition of STING-dependent signaling	Suppression of IFN-β production (in vitro)	[10]
Influenza virus	Mint3 depletion	-	AMPK activation	Attenuates severe pneumonia by influenza infection (in vivo)	[34]
	AICAR	Agonist	AMPK activation in Mint3 depletion model	Decreases inflammatory cytokine production in Mint3-deficient macrophages (in vitro)	[34]
	Curcumin	Activator	AMPK activation	Inhibits influenza A virus infection (in vitro and in vivo)	[36]
Human immunodeficiency virus-1	Epigallocatechin gallate	Activator	AMPK activation	Attenuation of Tat-induced human immunodeficiency virus 1 (HIV-1) transactivation	[37]
Human adenovirus	Adenovirus	-	Inhibit AMPK activity/signaling	Virus induces lipid droplets, presumably associated with obesity (in vitro)	[38]
Rift Valley Fever Virus (RVFV)	A769662, 2-deoxy-d-glucose (2-DG)	Agonist	LKB1/AMPK signaling activation; Inhibition of fatty acid synthesis	Restriction of viral infection (in vitro)	[39]
Herpes simplex virus type 1 (HSV-1)	AICAR, Resveratrol, Quercetin	Activator/agonist	AMPK/Sirt1 activation	Reduces viral titer and the expression of viral genes (in vitro)	[40]
Coxsackievirus B3 (CVB3)	-	-	AMPK activation by CVB3	Restriction of viral replication; reversed by siRNA against AMPK	[41]
	AICAR, A769662, Metformin	Agonist	AMPK activation	Restriction of viral replication; improve the survival rate of infected mice (in vitro and in vivo)	[41]
Epstein-Barr virus (EBV)	LMP1 of EBV	-	LKB1-AMPK inactivation	AMPK inactivation leads to proliferation and transformation of epithelial cells associated with EBV infection (in vitro)	[42]
	AICAR	Agonist	AMPK activation	Inhibition of proliferation of nasopharyngeal epithelial cells (in vitro)	[42]
Kaposi’s sarcoma-associated herpesvirus (KSHV)	AICAR, Metformin, Constitutive active AMPK	Agonist	AMPK as a KSHV restriction factor	Inhibits the expression of viral lytic genes and virion production (in vitro)	[43]
	Compound C, Knockdown of AMPKα1	Antagonist	AMPK inhibition	Enhances viral lytic gene expression and virion production (in vitro)	[43]

**Table 2:** Detrimental Effects of AMPK in viral infection.

Pathogen	Small Molecules/Chemicals	Agonist/Antagonist	Involvement of AMPK	Outcome (In Vitro/In Vivo)	Ref.
Rotavirus	RNAi	-	AMPK-mediated glycolysis, fatty acid oxidation and autophagy	Development of a rotavirus replication-permissive environment (in vitro)	[44]
	AICAR, Metformin	Agonist	AMPK activation (AICAR, directly; Metformin, indirectly)	Upregulation of the proportion of viral infected cells (in vitro)	[44]
	Dorsomorphin	Inhibitor	Inhibition of AMPK activity	Reduces the number of infected cells (in vitro)	[44]
Dengue virus	Virus infection	-	Elevates 3-hydroxy-3-methylglutaryl-CoA reductase activity through AMPK inactivation	Promotes the formation of viral replicative complexes (in vitro)	[45]
	Metformin, A769662	Agonist	AMPK activation	Antiviral effects (in vitro)	[45]
	Compound C	Antagonist	AMPK inhibition	Augments the viral genome copies (in vitro)	[45]
	Virus infection	-	AMPK activation; induction of lipophagy	Increases viral replication (in vitro)	[46]
	Compound C siRNA against AMPKα1	Antagonist	Inhibition of proviral lipophagy	Decreases viral replication (in vitro)	[46]
Hepatitis B virus (HBV)	HBx protein	-	Decreased ATP, activates AMPK in rat primary hepatocytes	AMPK inhibition decreases HBV replication (in vitro)	[47]
	Compound C	Antagonist	Activates mTORC1	Reduces HBV replication (in vitro)	[47]
Ebola virus	Compound C	Antagonist	Less permissive to Ebola virus infection (Similar effects in AMPKα1- or AMPKα2-deleted mouse embryonic fibroblasts)	Inhibits EBOV replication in Vero cells (in vitro)	[48]
Avian reovirus	Virus infection	-	Upregulates AMPK phosphorylation leading to p38 MAPK activation	Increases virus replication (in vitro)	[49]
	P17 protein	-	P17 protein activates AMPK to induce autophagy	Increases virus replication (in vitro)	[50]
	AICAR	Agonist	AMPK activation (Indirect effects through p38 MAPK)	Increases virus replication (in vitro)	[49]
	Compound C	Antagonist	AMPK inhibition	Decreases virus replication (in vitro)	[49]
Herpes simplex virus type 1 (HSV-1)	HSV-1	-	In early infection, AMPK is down-regulated, and then recovered gradually	AMPK/Sirt1 axis inhibits host apoptosis in early infection (in vitro)	[51]
Human immunodeficiency virus-1 (HIV1)	Cocaine	-	Induces AMPK upregulation; AMPK plays a role in energy deficit and metabolic dysfunction	Cocaine exposure during HIV infection accelerates neuronal dysfunction (in vitro)	[52]
Human cytomegalovirus (HCMV)	RNAi	-	AMPK may activate numerous metabolic pathways during HCMV infection	siRNA to AMPKα reduces HCMV replication (in vitro)	[53,54]
	HCMV	-	Upregulation of host AMPK	Favors viral replication (in vitro)	[53,54]
	Compound C	Antagonist	Interferes with normal accumulation of viral proteins and alters the core metabolism	Compound C inhibits the viral production of HCMV (in vitro); blocks the immediate early phase of viral replication (in vitro)	[53,54]
	RNAi to AMPK	-	Blocks glycolytic activation in HCMV-infected cells	RNA-based inhibition of AMPK attenuates HCMV replication (in vitro)	[55]
	Digitoxin	Activator	Digitoxin modulates AMPK-ULK1 and mTOR activity to increase autophagic flux	Viral inhibition (in vitro)	[56]
	Digitoxin + AICAR	-	Combination reduces autophagy	Viral replication (in vitro)	[56]
	HCMV	-	Induces targeting host protein viperin to mitochondria; viperin is required for AMPK activation and regulate lipid metabolism	Viperin-dependent lipogenesis promotes viral replication and production by infected host cells (in vitro)	[57]
Respiratory syncytial virus (RSV)	RSV	-	RSV induces autophagy through ROS and AMPK activation	RSV-induced autophagy favors viral replication (in vitro)	[59]
	Compound C	Antagonist	Inhibition of AMPK and autophagy	Compound C reduces viral gene and protein expression, and total viral titers (in vitro)	
Bluetongue virus	Bluetongue virus	-	Induces autophagy through activation of AMPK	Favors viral replication (in vitro)	[60]
	Compound C siRNA to AMPK	Antagonist	Inhibits BTV1-induced autophagy	AMPK inhibition decreases viral titers (in vitro)	
Rabies virus	Rabies virus		Incomplete autophagy induction via CASP2-AMPK-MAPK1/3/11-AKT1-mTOR pathways	Enhances viral replication (in vitro)	[61]
Sendai virus	Sendai virus	-	Induces host protein TDRD7, an inhibitor of autophagy-inducing AMPK	Host autophagy and viral replication is inhibited by TDRD7 (in vitro)	[62]
	Compound C, shRNA to AMPK	Antagonist	Inhibition of AMPK activity; inhibits viral protein	AMPK activity is required for viral replication (in vitro)	
Snakehead vesiculo-virus	Snakehead vesiculo-virus	-	Downregulates miR-214, which targets AMPK	AMPK upregulation promotes viral replication through reduction of IFN-α expression (in vitro)	[65]

**Table 3:** The roles of AMPK in bacterial infection.

Pathogen	Small Molecules/Chemicals	Agonist/Antagonist	Involvement of AMPK	Outcome (In Vitro/In Vivo)	Ref.
*L. pneumophila*	-	-	Chronic AMPK activation involved in host susceptibility to infection (Direct effects by AMPKα antisense)	Bacterial multiplication in host cells with mitochondrial dysfunction	[66]
	Metformin	Agonist	Bactericidal effects are mediated by mitochondrial ROS production (Indirect)	Antimicrobial responses (in vitro and in vivo)	[67]
*S. typhymurium*	-	-	*S. typhymurium* exhibits virulence through lysosomal degradation of SIRT1 and AMPK to impair autophagy	Bacterial evasion from autophagic clearance (in vitro)	[68]
	AICAR	Agonist	Upregulation of autophagy	Increased colocalization of salmonella containing vacuole with LC3 (in vitro)	[68]
	-	-	AMPK activation via TAK1; autophagy initiation by ULK1 phosphorylation	Autophagy activation (in vitro)	[69]
	Compound C	Antagonist	AMPK inhibition	Increased bacterial replication by suppression of autophagy (in vitro)	[69]
*B. abortus*	-	-	AMPK activation via inositol-requiring enzyme 1 (IRE1)	Promote intracellular growth of *B. abortus* (in vitro)	[72]
	Compound C	Antagonist	AMPK inhibition; activation of NADPH oxidase-mediated ROS production	Suppression of intracellular growth (in vitro)	[72]
*E. coli*	Piperine	Antagonist	Inhibits ATP-induced pyroptosis by suppressing AMPK activation	Inhibition of pyroptosis; Attenuation of systemic inflammation (in vitro and in vivo)	[73]
	ATP Metformin	Agonist	AMPK activation; increases pyroptosis by inflammasome activation	Activation of pyroptosis (in vitro)	[73]
*B. thuringiensis*	-	-	AMPK identified by transcriptome and proteome data analysis in vivo (Indirect)	Potentially related to regulation of immune defense (Not determined)	[74]
*H. pylori*	-	-	TAK1-mediated AMPK activation	Protects gastric epithelial cells from *H. pylori*-induced apoptosis (in vitro)	[75]
	A-769662 Resveratrol	Agonist	Inhibits *H. pylori*-induced apoptosis (Direct effects by overexpression of AMPKα)	Alleviates *H. pylori*-induced gastric epithelial cell apoptosis (in vitro)	[76]
	Compound 13	Agonist	Inhibits *H. pylori*-induced apoptosis through AMPK-heme oxygenase-1 signaling	Alleviates *H. pylori*-induced gastric epithelial cell apoptosis (in vitro)	[76]
	Compound C	Antagonist	Inhibitory effects upon compound 13-mediated anti-*H. pylori* activities (Direct effects by AMPKα1 shRNAs)	Aggravates *H. pylori*-induced gastric epithelial cell apoptosis (in vitro)	[76]
*P. aeruginosa*	AICAR Metformin	Agonist	Counteracts the bacterial effects on the reduction of transepithelial electrical resistance (Indirect effects)	Inhibits hyperglycemia-induced bacterial growth; Improve airway epithelial barrier function (in vitro)	[77]
*S. aureus*	AICAR	Agonist	AMPK activation	Reduces bacterial burden and intraocular inflammation; Increases bacterial killing in macrophages (in vitro and in vivo)	[78]
	Compound C	Antagonist	Downregulates AMPK activity (Direct effects by AMPKα1 knockout mice)	Counteracts AICAR-mediated anti-inflammatory effects (in vivo); Increases susceptibility towards *S. aureus* endophthalmitis (in vivo)	[78]
*P. acne*	Epigallocatechin gallate	-	Activates AMPK-sterol regulatory element-binding proteins pathway activation	Antilipogenic effects in SEB-1 sebocytes (in vitro)	[35]
*A. baumannii*	-	-	Activates autophagy through Beclin-1-dependent AMPK/ERK/mTOR pathway (Indirect effects by different *A. baumannii* strains)	Autophagy may promote antimicrobial responses (in vivo)	[79]
*Mycobacterium tuberculosis*	Metformin	Agonist	AMPK activation; Increased mtROS production; Increases phago-lysosomal fusion (Direct effects upon bacterial growth in vitro)	Inhibition of intracellular growth of *M. tuberculosis* (drug-resistant strain; in vitro); Increases the efficacy of conventional TB drugs in vivo	[80]
	AICAR	Agonist	AMPK-PPARGC1A signaling-mediated autophagy activation; Enhancement of phagosomal maturation (Direct effects by shRNA against AMPKα)	Upregulation of antimicrobial responses (in vitro and in vivo)	[12]
	Compound C	Antagonist	Counteracts the effects by AICAR upon intracellular inhibition of *M. tuberculosis* growth	Downregulation of antimicrobial responses (in vitro)	[12]
	Vitamin D (1,25-D3)	-	Induces autophagy through LL-37 and AMPK activation (Indirect effects upon LL-37 function)	Promotes autophagy and antimicrobial response in human monocytes/macrophages (in vitro)	[81]
	Phenylbutyrate Vitamin D	-	Induces LL-37-mediated autophagy (Indirect effects; AMPK is involved in LL-37-mediated autophagy)	Improves intracellular killing of *M. tuberculosis* (in vitro)	[82]
	Gamma-aminobutyric acid (GABA)	Agonist	Induces autophagy (Direct effects by shRNA against AMPK)	Promotes antimicrobial effects against *M. tuberculosis* (in vitro and in vivo)	[83]
	Ohmyungsamycins	-	Activates AMPK and autophagy; Intracellular inhibition of bacterial growth; Amelioration of inflammation (Indirect effects upon host autophagy)	Promotes antimicrobial effects against *M. tuberculosis* (in vitro and in vivo)	[26]
	Compound C	Antagonist	Blocks the secretion of neutrophil Matrix metalloproteinase-8 (MMP-8)	Neutrophil MMP-8 secretion is related to matrix destruction in human pulmonary TB (in vitro and in human TB lung specimens)	[84]

**Table 4:** The role of AMPK in parasitic infection.

Pathogen	Small Molecules/Chemicals	Agonist/Antagonist	Involvement of AMPK (Direct/Indirect)	Outcome (In Vitro/In Vivo)	Ref.
Hookworm *Nippostrongylus brasiliensis*	-		AMPKα1 deficiency inhibit IL-13 and CCL17, and defective type 2 immune resistance (Direct effects using by AMPKα1 knockout mice)	AMPKα1 suppresses lung injury and drives M2 polarization during infection	[89]
*L. infantum*	-	-	Infection leads to a metabolic switch to activate AMPK through the SIRT1-LKB1 axis (Direct effects using by AMPKα1 knockout mice)	Ablation of AMPK promotes parasite clearance in vitro and in vivo	[90]
*S. japonicum*			Infection-driven IL-7-IL-7R signaling inhibits autophagy; IL-7 inhibits macrophage autophagy via AMPK	Anti-autophagic IL-7 increases liver pathology (in vivo)	[91]
	Metformin	Agonist	Decreases the autophagosome formation in macrophages	in vitro	
	Compound C siAMPKα	Antagonist	Increases autophagosome formation in macrophages (Direct effects by siAMPKα)	in vitro	
*P. falciparum*	-	-	AMPK activity is suppressed upon infection	Decreases *Plasmodium* hepatic growth	[92]
	Salicylate Metformin A769662	Agonist	AMPK activation impairs the intracellular replication of malaria	Antimalarial interventions (in vitro and in vivo)	
*Trypanosoma cruzi*	Resveratrol, Metformin	Agonist	AMPK activation reduces heart oxidative stress (Indirect effects)	Reduces heart parasite burden; Protects heart function in Chagas heart disease (in vivo)	[93]

## Abbreviations

ADP	Adenosine diphosphate
AICAR	5-aminoimidazole-4-carboxamide 1-β-d-ribofuanoside
AMP	Adenosine monophosphate
AMPK	5′-AMP-activated protein kinase
ATF3	Activating transcription factor 3
ATP	Adenosine triphosphate
CaMKK	Ca2+/calmodulin-dependent protein kinase kinases
CCL17	Chemokine ligand 17
CVB3	Coxsackie virus B3
DM	Diabetes mellitus
EBOV	Zaire Ebolavirus
EBV	Epstein-Barr Virus
GABA	Gamma-aminobutyric acid
HBV	Hepatitis B virus
HCC	Hepatocellular carcinoma
HCMV	Human cytomegalovirus
HCV	Hepatitis C virus
HIV	Human immunodeficiency virus
HMGCR	3-hydroxy-3-methylglutaryl-CoA reductase
HSV-1	Herpex Simplex Virus Type 1
IFN	Interferons
IL-17	Interleukin-17
IL-1β	Interleukin-1β
IL-37	Interleukin-37
IL-7	Interleukin-7
KSHV	Kaposi’s sarcoma-associated herpesvirus
LKB1	Liver kinase B1
LMP1	Latent membrane protein 1
MAPK	Mitogen-activated protein kinase
MMP-8	Matrix metalloproteinase-8
mTOR	Mammalian target of rapamycin
mTORC1	Mammalian target of rapamycin complex 1
NADPH	Nicotinamide adenine dinucleotide phosphate
NF-κB	Nuclear factor kappa-light-chain-enhancer of activated B cells
PPARGC1A	Peroxisome proliferator-activated receptor-gamma, coactivator 1α
ROS	Reactive oxygen species
RVFV	Rift Valley Fever Virus
SIRT1	Sirtuin 1
SIV	Simian immunodeficiency virus
SNARK	Sucrose-non-fermenting protein kinase 1/AMP-activated protein kinase-related protein kinase
STING	Stimulator of IFN genes
TAK1	Transforming growth factor (TGF)-β-activated kinase 1
TLR	Toll-like receptor
TSC	Tuberous sclerosis complex
